# A Rare Outcome for Transient Parotitis After Upper Gastrointestinal Endoscopy: A Case Report and Brief Review of the Literature

**DOI:** 10.7759/cureus.65374

**Published:** 2024-07-25

**Authors:** Fathima Nilofar, Mohamed Bilal Azam

**Affiliations:** 1 General Medicine, Saveetha Medical College and Hospital, Chennai, IND; 2 Medical Gastroenterology, Saveetha Medical College and Hospital, Chennai, IND

**Keywords:** symptomatic treatment, warm compression, post endoscopy complication, analgesics, upper gastrointestinal endoscopy, transient parotitis

## Abstract

Upper gastrointestinal (GI) endoscopy, though generally safe, can rarely cause complications such as transient parotitis, which typically resolves within 24 hours. Parotitis may occur due to salivary duct blockage, venous congestion from straining, or reflex parasympathetic stimulation. We discuss a 33-year-old chronic alcoholic man who developed right parotid gland swelling immediately following an upper GI endoscopy, conducted without sedation to evaluate his epigastric pain, vomiting, anorexia, and weight loss. His blood tests and abdominal ultrasound were normal. Patient developed sharp pain and swelling in the right parotid gland post-procedure. An ultrasound revealed diffuse gland swelling without abscess or lymph node enlargement. He was treated with analgesics, warm compresses, and a semisolid diet, leading to symptom resolution within 12 hours. Post-endoscopy transient parotitis is rare and typically benign, with limited evidence from case reports and small series. Diagnosis through ultrasound is crucial to exclude other causes, and treatment is mainly symptomatic, involving analgesics and warm compresses, with antibiotics if infection is suspected. This case report and brief review of literature underscore the self-limiting nature of transient parotitis following endoscopy.

## Introduction

Upper gastrointestinal (GI) endoscopy is safe, and complications such as parotitis rarely occur [1]. Parotid gland swelling is caused by the inflammation and enlargement of the parotid gland. The most common causes of parotitis are viral or bacterial infections, mechanical trauma, and ductal obstruction.

Post-endoscopy parotitis is a transient complication that subsides within 24 hours. This complication has also been reported to occur after bronchoscopy and endotracheal intubation [2]. It is most important to note that this complication is the transient nature of symptoms and spontaneous remission happens within a few days. The mechanisms underlying post-endoscopy transient parotitis are blockage of salivary ducts by the increased secretions during endoscopy, venous congestion caused by straining or coughing during the procedure [3], and reflex parasympathetic stimulation during the procedure leading to vasodilation of the parotid gland [4]. Typically, the parotid and submaxillary glands are involved. Prolonged endoscopy is considered a risk factor for post-endoscopy transient parotitis.

Local ultrasonography should be conducted for every patient. To address the transient parotitis, analgesics, warm compression, and iodine gargles may be used. A short course of antibiotics may also be prescribed if the patient develops a fever. If the parotitis does not resolve rapidly, computed tomography should be used to rule out other etiology of abscess/calcifications and ductal calculi.

Here, we report the case of a young man who developed parotid swelling shortly after upper GI endoscopy. This case report contributes to the limited evidence of post-endoscopy parotid swelling, raising awareness among clinicians and optimizing management strategies.

## Case presentation

A 33-year-old man with chronic alcoholism presented with complaints of burning epigastric pain for three months that gradually initiated but aggravated after food intake and was partially alleviated after treatment with proton pump inhibitors. The patient also experienced vomiting, anorexia, and loss of weight. No history of fever was documented, and blood investigations were normal (Table 1).

**Table 1:** Laboratory measurements of blood parameters

Parameter	Value	Normal Range
Hemoglobin	13.9	12-15 g/dL
Platelet Count	2.98	1.5-4.0 (x 10^6^ /mm^3^ )
Leukocyte count	5390	4000-10,000 (cells/ mm^3^ )
International normalized ratio	0.9	<1.1
Serum albumin	3.8	3.5-5.0 (g/dL)
Serum amylase	88	<100 (IU/L)
Serum lipase	66	23-300 (IU/L)

Ultrasonography of the abdomen showed no significant abnormality. Serology results were negative. To rule out acid peptic disease, the patient was scheduled for an upper GI endoscopy. The procedure was explained to the patient, and the patient’s consent was obtained. Before the procedure, 2% lignocaine spray was administered. The procedure was conducted without any sedation administered to the patient. The total duration of the procedure was seven minutes.

**Figure 1:**
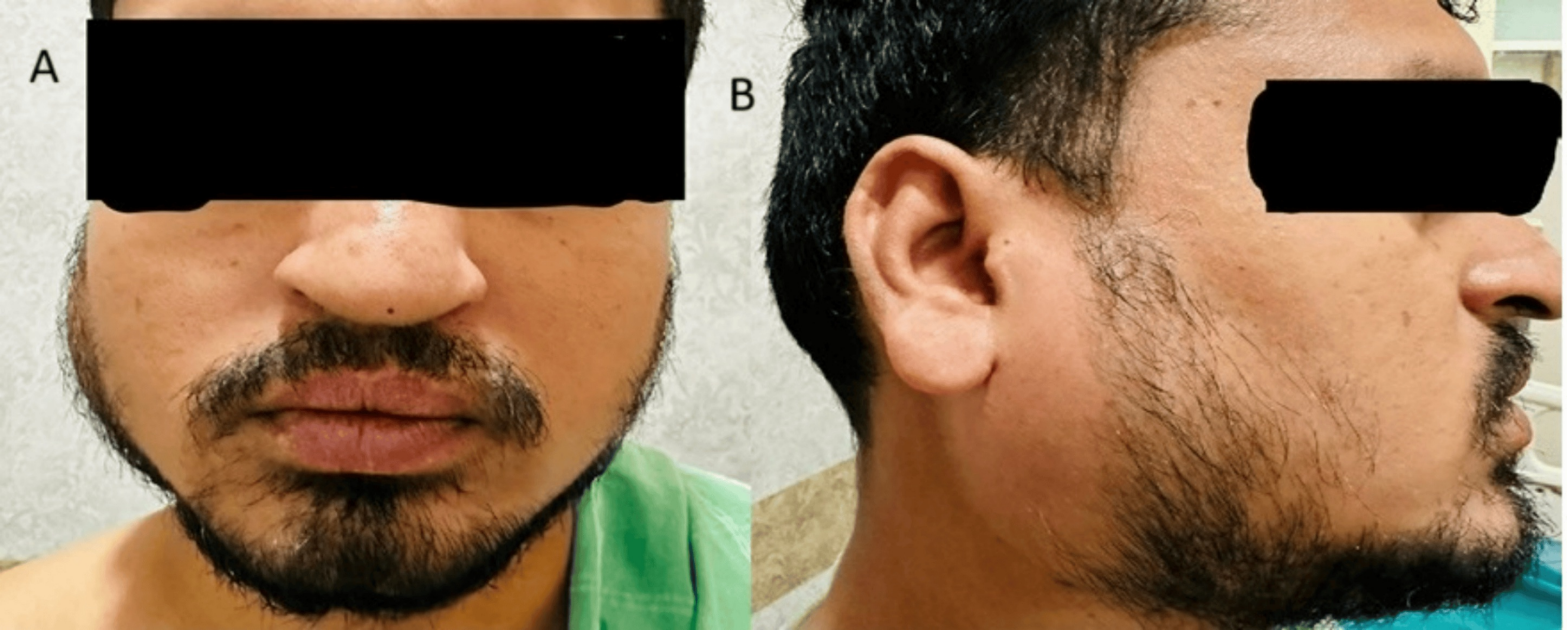
Front (A) and side (B) views of the swelling in the right parotid gland

A flexible video endoscope was used for the procedure. The results revealed pre-pyloric ulcers and erosions in the D1 segment. A biopsy was made at the antrum and tested for H. pylori. A few minutes after the procedure, the patient developed swelling in the right parotid gland (Figure 1) and sudden pricking pain that radiated to the jaw. The pain aggravated upon opening the mouth and chewing. The patient did not develop any fever.

Local examination revealed erythematous swelling palpable in the right parotid region, which was tender on touch with no warmth, suggestive of parotitis. There was no crepitus, and the patient was hemodynamically stable.

**Figure 2:**
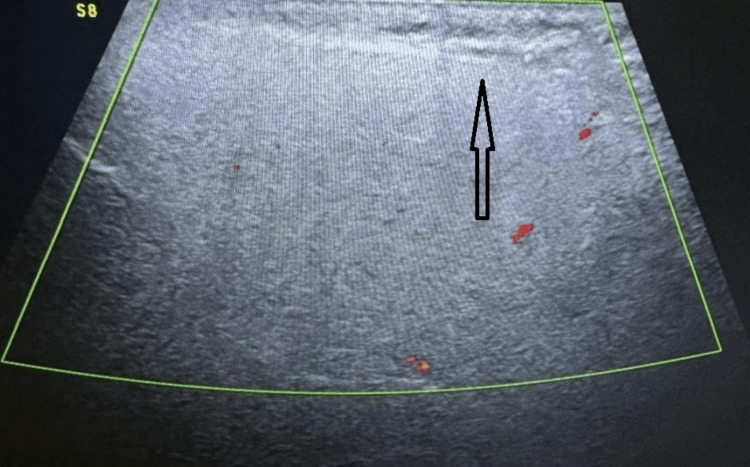
Ultrasonography of the swollen right parotid gland Black arrow represents swelling in the right parotid gland

**Figure 3:**
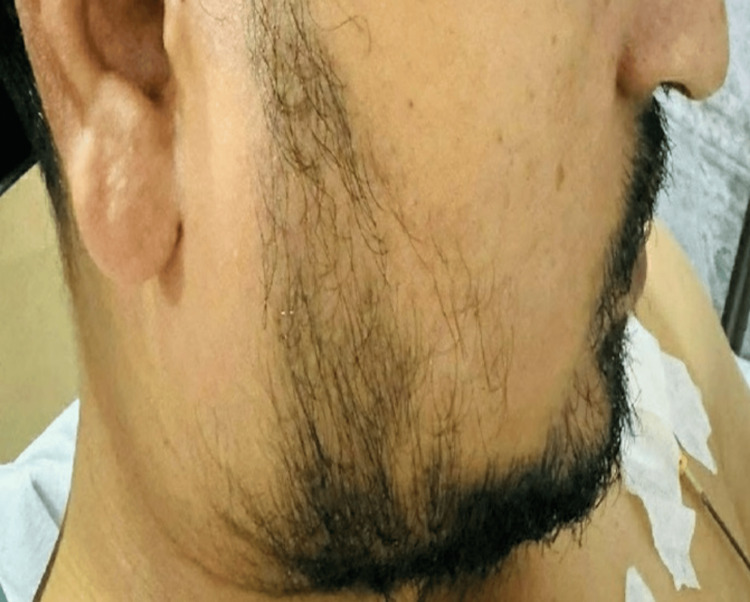
Side view showing no swelling of the right parotid gland 12 hours after the endoscopy

Ultrasonography of the swollen parotid gland revealed diffuse swelling without any increase in vascularity (Figure 2). There was no abscess formation, fluid collection, or lymph node enlargement. To manage the swelling, the patient was prescribed an intravenous analgesic (tramadol) and provided warm compression. The patient was asked to consume only semisolid diet. The swelling subsided within 12 hours (Figure 3) with complete relief from pain. The patient was closely monitored for two days and then discharged.

## Discussion

Post-endoscopy transient parotitis is an uncommon but recognized complication of upper GI endoscopy. The exact incidence rate is not well documented due to its rarity, and the available literature primarily comprises case reports and small case series (Table 2).

**Table 2:** . Review of literature for case reports and small case series u/l-Unilateral b/l-Bilateral

Author (citation)	Year	Duration of procedure	Indication for the procedure	Site of swelling (u/l or b/l)	Remedial measures taken
Taori K et al. [5]	2024	8 minutes	Known case of cirrhosis with portal hypertension with black colored stools for 2 weeks	Right parotid gland	Warm compression Close monitoring for 48 hours
Lamtha SC et al. [6]	2019	3 minutes	Dyspepsia	Right parotid gland	Swelling subsided on its own within 4 hours
Can G et al. [7]	2014	4 minutes	Status post total gastrectomy with dysphagia	Bilateral parotid glands	Swelling subsided 3 hours after hydration treatment
Ziccardi V et al. [8]	1992	2 minutes	Hematemesis	Marked bilateral swelling at mandibular angle and mild periorbital edema	Swelling subsided on its own within 2 hours
Marvin JG [9]	1976	2 minutes	Recurrent abdominal pain for 2 months	Right Parotid gland	Swelling subsided on its own within 30 minutes
Kumaran SV et al. [1]	2013	2 minutes	Non-ulcer dyspepsia	Right Parotid gland	Swelling subsided on its own within 3 hours
Nijhawan S [3]	1992	Immediately	Gastrointestinal bleeding	Left parotid gland	Swelling subsided gradually within 4 hours

Several mechanisms have been proposed to explain this condition. One hypothesis is the mechanical blockage of salivary ducts by the increased secretions during endoscopy, which leads to swelling and inflammation of the parotid gland. Another possibility is venous congestion caused by straining or coughing during the procedure. Some have also suggested reflex parasympathetic stimulation that leads to vasodilation of the parotid gland as a cause of post-endoscopy transient parotitis.

Patients typically present with a sudden onset of parotid swelling and pain shortly after endoscopy, as seen in the present case. The transient nature and spontaneous resolution of symptoms within 24 hours are characteristic features of this condition. Diagnostic imaging, such as ultrasound imaging, is crucial for ruling out other causes of parotid swelling, such as abscesses, fluid collection, or ductal calculi.

The management of post-endoscopy transient parotitis primarily involves symptomatic treatment. Analgesics, such as tramadol, provide relief from pain. Warm compression helps reduce swelling. A semisolid diet may minimize discomfort during eating. If the patient has fever or signs of infection, a short course of antibiotics may be necessary. In the present case, the patient’s symptoms resolved within 12 hours through conservative management strategies, consistent with the transient nature of the condition.

The reported evidence of post-endoscopy transient parotitis is limited. Kumaran et al. (2013) reported a case similar to ours, emphasizing the rarity and transient nature of the condition [1]. Nijhawan and Rai (1992) and Strowbridge (1987) have provided insights into mechanisms underlying the development of parotitis, supporting the hypotheses of ductal blockage, venous congestion, and parasympathetic stimulation [3,4].

## Conclusions

Transient parotitis is a rare but benign complication that can occur after upper GI endoscopy. Awareness of this potential complication is essential for clinicians to promptly recognize and manage the condition, thereby avoiding unnecessary diagnostic procedures and interventions. This case report contributes to the limited body of evidence of post-endoscopy transient parotitis and underscores the importance of considering this diagnosis in patients presenting with sudden parotid swelling after endoscopy.
